# Maternal parity modifies the association of birthweight polygenic score with fetal growth

**DOI:** 10.1038/s41598-025-10415-1

**Published:** 2025-07-31

**Authors:** Prabhavi Wijesiriwardhana, Tesfa Dejenie Habtewold, Guisong Wang, Jessica L. Gleason, Ronald J. Wapner, Katherine L. Grantz, Fasil Tekola-Ayele

**Affiliations:** 1 Epidemiology Branch, Division of Population Health Research, Division of Intramural Research, Eunice Kennedy Shriver National Institute of Child Health and Human Development, National Institutes of Health, Bethesda, MD 20892-7004 USA; 2 The Prospective Group (TPG), Institute of Child Health and Human Development, National Institutes of Health, Bethesda, MD 20892-7004 USA; 3 Department of Obstetrics and Gynecology, Columbia University, New York, NY 10032 USA

**Keywords:** Fetal growth, Birthweight, Parity, Pregnancy, Maternal genetic risk, Multi-ancestral, Developmental biology, Genetics, Medical research, Risk factors

## Abstract

Low birthweight is more common among children born to nulliparas (women with no prior pregnancy lasting ≥20 weeks of gestation) compared to those born to multiparas (women with one or more prior pregnancies lasting ≥20 weeks). We investigated whether parity modifies the association of maternal genetic risk score (mGRS) of maternal birthweight-reducing genetic variants with fetal size and weekly growth pace (i.e., change in fetal size over a 1-week gestational age interval) at gestational weeks 10–40 in two multi-ancestral cohorts of pregnant women. mGRS, derived from previously identified birthweight-reducing maternal variants was tested for association with fetal size and weekly growth pace using linear regression adjusted for fetal sex and top 10 genetic principal components. Among nulliparas, but not among multiparas, higher birthweight-reducing mGRS was associated with lower fetal size and slower weekly growth pace as measured by fetal weight, humerus and femur lengths, and abdominal and head circumferences beginning from gestational week 11. The findings suggest that the maternal genetic factors target major physiological changes at the first pregnancy that get less profound with multi-parity. Considering parity as a biological variable may facilitate the precision of identifying sensitive intrauterine periods and interventions for pregnancy outcomes using genomics.

**Supplementary Information:**

The online version contains supplementary material available at 10.1038/s41598-025-10415-1.

## Introduction

Optimal fetal growth is important for a healthy pregnancy outcome and for lifelong health of an individual^1–3^. Fetal growth is influenced by interactions of the maternal and fetal genomes with environmental factors^4–6^. Recent genome-wide association studies (GWAS) have estimated that the maternal genome accounts for approximately 7.6% of the heritability of birthweight and identified 37 maternal genetic loci that influence birthweight through the intrauterine environment^4,7^. However, birthweight does not reflect the trajectory and pace of fetal growth (i.e., change in fetal size over a given gestational age interval), measures which vary by gestational age and type of fetal biometry^8–13^. Furthermore, the genetic influence on fetal growth may also vary by gestational age^14–18^. Genomic insights on fetal biometry measures can inform intrauterine intervention targets at sensitive gestational ages; yet the timing of association of birthweight-reducing maternal genetic factors on fetal growth is unclear.

The maternal genome is likely to interact with various non-genetic factors in its influence on fetal growth through the intrauterine environment. Reproductive history such as parity, which represents the number of previous pregnancies lasting at least 20 weeks of gestation regardless of outcome^19–25^ has reproducibly been associated with fetal growth measures at birth. Children born to nulliparas (women with no prior pregnancy lasting ≥20 weeks of gestation) have lower birthweight, head circumference, length, ponderal index, and body mass index compared to those born to multiparas (women with one or more previous pregnancies lasting at least 20 weeks of gestation regardless of outcome)^20–25^. Studies of women with successive pregnancies revealed that birthweight is lower among nulliparas after accounting for within-woman factors that change with increasing parity such as maternal age^26^ pre-pregnancy weight and weight gain during pregnancy^27,28^ and medical conditions such as type 2 diabetes mellitus and hypertension^29–31^.

Although the mechanisms behind the association of nulliparity with lower birthweight are unclear, several studies have shown that nulliparous pregnancies are not as efficient as multiparous pregnancies in supporting fetal growth^32^. Nulliparous pregnancies have lower uteroplacental blood flow rate and uterine perfusion^32–35^ shorter endometrial cavity length and smaller uterine capacity^36–38^. Nulliparous women lack pregnancy-related immunological memory that contributes to improved immunologic tolerance to the allogenic fetus and reduced risk of fetal growth restriction^39–41^. These observations suggest that adaptive physiological changes in pregnancy are less developed during the first pregnancy, potentially underlying the link between nulliparity and lower fetal growth^42^.

Maternal genes associated with birthweight have been implicated in phenotypes related to cardiovascular (e.g. *SH2B3* gene^43^), hemostasis/coagulation (e.g. *SLC25A37* gene^44,45^), and immune (e.g. *ZBTB38* gene^46^) systems. This convergence of biological processes impacted by birthweight-related maternal genes and by parity suggests a potential relationship between those genes and parity-related cardiovascular, immunologic, and uteroplacental changes in influencing fetal growth. These insights can facilitate the precision of interventions for promoting fetal growth and subsequent health outcomes. However, to date, there is no evidence on whether birthweight-related maternal genetic influence on fetal growth varies by parity. In this study, we investigated the association of genetic risk score of maternal birthweight-reducing genetic variants (mGRS) with fetal size and weekly growth pace at gestational weeks 10–40 by maternal parity.

## Results

The discovery analysis was done using data from 822 nulliparous and 981 multiparous pregnant women recruited through the NICHD Fetal Growth Studies-Singletons^47^ and the replication analysis for estimated fetal weight was performed using data from 6058 nulliparous pregnant women recruited through the Nulliparous Pregnancy Outcome Study: Monitoring Mothers-to-be (nuMoM2b)^48^. In the discovery cohort, nulliparas had significantly lower maternal age (mean ± SD: 26.6 ± 5.4 vs. 29.2 ± 5.3 years), pre-pregnancy BMI (24.7 ± 4.9 vs. 26.2 ± 5.5 kg/m^2^), and birthweight (3258 ± 472 vs. 3330 ± 463 g), and had higher proportion of pregnancy-related hypertensive disorders (10.22% vs. 4.89%) and infants born small-for-gestational age (10.83% vs. 7.44%) compared to multiparas. The ancestry distributions of the nulliparas were 10.9% East Asian American, 21.3% Hispanic American, 31.1% African American and 31.5% European American, and that of multiparas was 10.6% East Asian American, 32.9% Hispanic American, 29.3% African American and 27.2% European American. The participants in the replication cohort (*n* = 6042 nulliparas) had mean ± SD age of 27.1 ± 5.6 years, pre-pregnancy BMI of 25.5 ± 6.1 kg/m^2^, and birthweight of 3305 ± 528 g. The ancestry distribution of the women in the replication cohort was 19.0% Hispanic American, 13.5% African American, and 67.5% European American. mGRS was derived from previously identified birthweight-reducing maternal variants^4,7^. The mean ± SD of mGRS was 0.89 ± 0.11 and 0.91 ± 0.11 units in the discovery and replication cohorts, respectively (Table 1). Most (29/33) SNPs in the mGRS were significantly correlated with at least one fetal growth biometry measure (Supplementary Table 2).

**Table 1:** Characteristics of study participants in the discovery and replication cohorts.

Characteristics	Discovery dataset (NICHD Fetal Growth Studies Singletons cohort)			Replication dataset (Nulliparous Pregnancy Outcomes Study: Monitoring Mothers-to-Be) (*n* = 6042)
	Nulliparous women (*n* = 822)	Multiparous women (*n* = 981)	*p*-value	
Maternal age (years), mean (SD)	26.6 (5.4)	29.2 (5.3)	**< 0.001**	27.1 (5.6)
Ancestry group, n (%)			**< 0.001**	
East Asian American	90 (10.95%)	104 (10.6%)		N/A
Hispanic American	175 (21.29%)	323 (32.93%)		1149 (19.02%)
African American	256 (31.14%)	287 (29.26%)		813 (13.46%)
European American	301 (36.62%)	267 (27.22%)		4080 (67.53%)
Maternal pre-pregnancy BMI (kg/m^2^), mean (SD)	24.7 (4.9)	26.2 (5.5)	**< 0.001**	25.5 (6.1)
Smoked during pregnancy, n (%)	4 (0.49%)	4 (0.41%)	0.54	344 (5.7%)
Gestational diabetes mellitus, n (%)	9 (1.09%)	17 (1.73%)	0.35	114 (1.9%)
Pregnancy-related hypertensive disorders, n (%)	84 (10.22%)	48 (4.89%)	**< 0.001**	346 (5.7%)
Gestational age at delivery, mean (SD)	39.35 (1.75)	39.12 (1.71)	**0.006**	38.91 (1.77)
Fetal sex, n (%)			0.92	
Male	417 (50.73%)	501 (51.07%)		3112 (51.51%)
Female	405 (49.27%)	480 (48.93%)		2928 (48.46%)
Birthweight (g), mean (SD)	3258 (472)	3330 (463)	**0.001**	3305 (528)
Small-for-gestational age (birthweight < 10th percentile*), n (%)	89 (10.83%)	73 (7.44%)	**0.015**	651 (10.8%)
Genetic risk score (GRS z-score), mean (SD)	0.89 (0.11)	0.89 (0.11)	0.99	0.91 (0.11)

*Based on the birthweight reference by Duryea et al.^49^.

Significant values (*p*-value <0.05) are in bold.

### Association between mGRS and fetal growth measures across gestation weeks 10–40

**Fig. 1:**
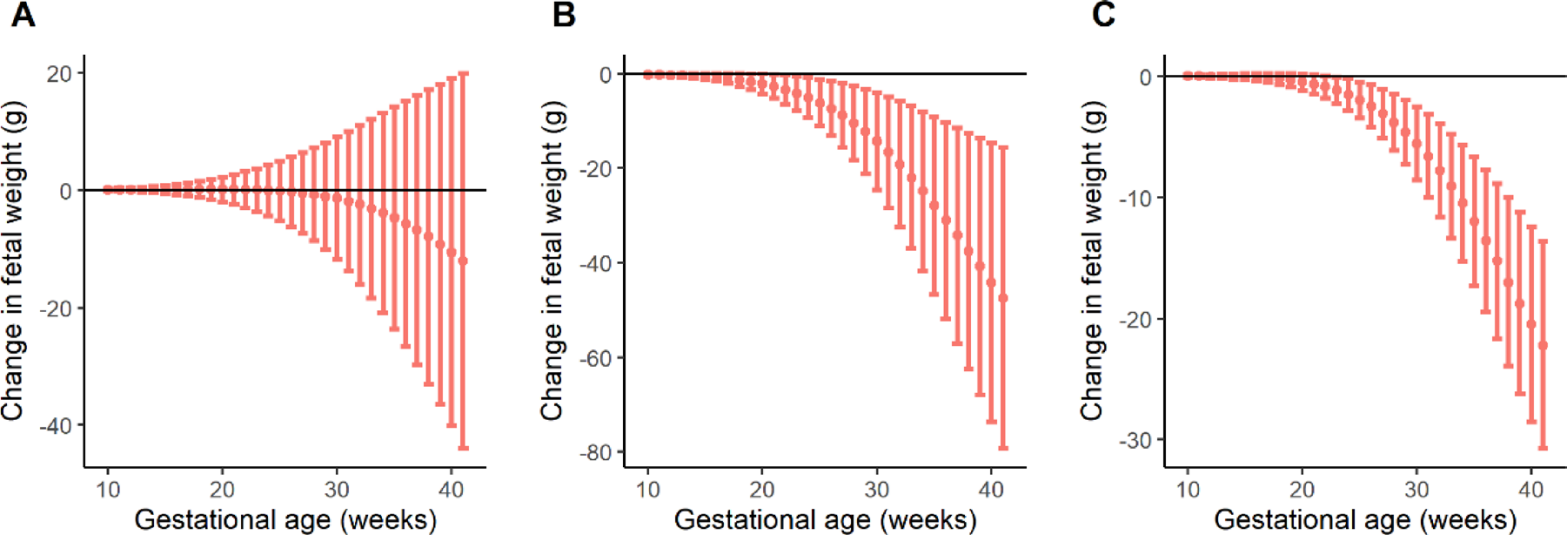
Change in estimated fetal weight (g) per 1-standard deviation (SD) increase in maternal genetic risk score (z-mGRS) of birthweight-lowering variants. (**A**) Multiparas in NICHD Fetal Growth Studies. (**B**) Nulliparas in NICHD Fetal Growth Studies. (**C**) Nulliparas in Nulliparous Pregnancy Outcomes Study: Monitoring Mothers-to-Be cohort.

Among nulliparas in the discovery dataset, birthweight-lowering mGRS was significantly associated with lower EFW from 20 to 40 gestational weeks (change in EFW at week 20 per standard deviation increase in mGRS: -2.15, 95% CI: -4.23, -0.06 g; change in EFW at week 40 per standard deviation increase in mGRS: -44.11, 95% CI: -73.60, -14.63 g) but was not significant among multiparas. The replication analysis among nulliparas in the nuMoM2b cohort similarly showed that birthweight-lowering mGRS was associated with lower EFW from 23 to 40 weeks (change in EFW at week 23 per standard deviation increase in mGRS: -1.16, 95% CI: -2.27, -0.04 g; change in EFW at week 40 per standard deviation increase in mGRS: -22.53, 95% CI: -33.86, -11.20 g) (Fig. 1, Supplementary Table 3).

Birthweight-lowering mGRS was also significantly associated with lower HC, AC, HL, and FL among nulliparas in the discovery cohort beginning as early as the first trimester (week 22 for HC, week 28 for AC, week 12 for HL, and week 17 for FL), but no significant associations were found among multiparas (Supplementary Table 4).

### Association between mGRS and weekly fetal growth Pace across gestation weeks 11–40

**Fig. 2:**
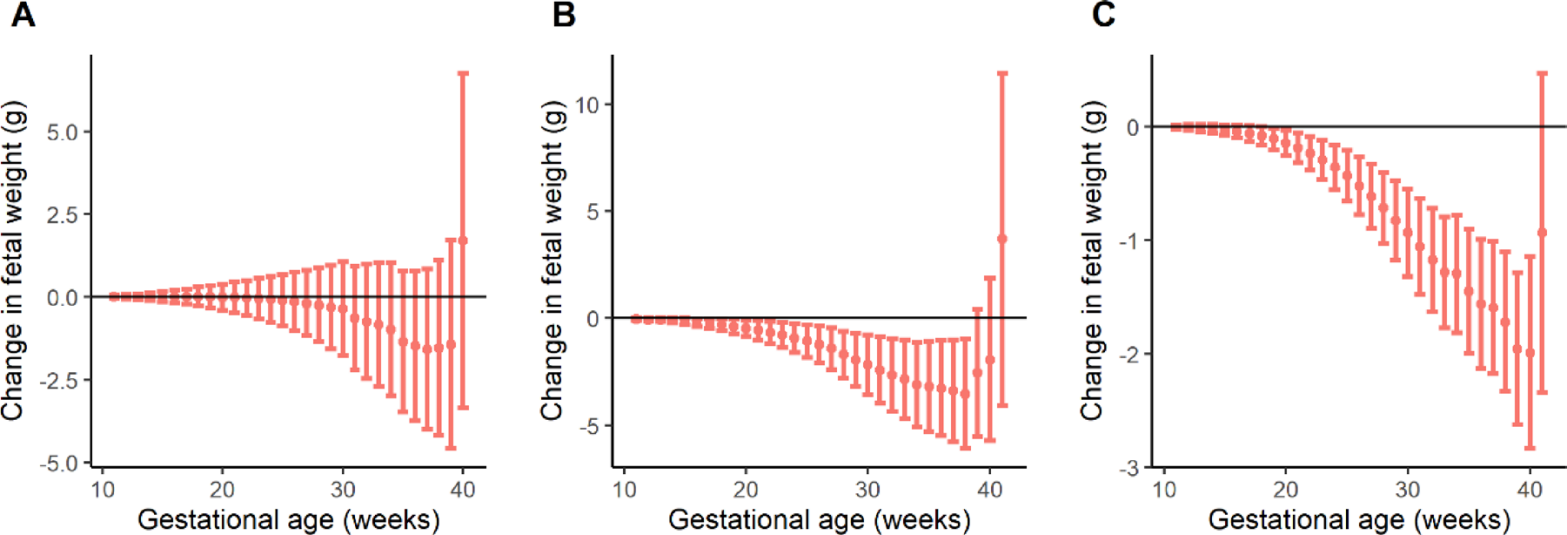
Change in weekly pace of estimated fetal weight (g) per unit 1-standard deviation (SD) increase in maternal genetic risk score (z-mGRS) of birthweight-lowering variants. (**A**) Multiparas in NICHD Fetal Growth Studies. (**B**) Nulliparas in NICHD Fetal Growth Studies. (**C**) Nulliparas in Nulliparous Pregnancy Outcomes Study: Monitoring Mothers-to-Be cohort.

Among nulliparas, birthweight-lowering mGRS was associated with slower weekly EFW growth pace from 16 to 38 gestational weeks (at week 16: -0.19, 95% CI: -0.37, -0.00 g/week; at week 38: -3.53, 95% CI: -6.09, -0.97 g/week) but no significant association was found in multiparas. In the replication dataset of nulliparas, the association of mGRS with slower weekly EFW growth pace from 19 to 40 weeks (at week 19: -0.11, 95% CI: -0.20, -0.01 g/week; at week 40: -2.00, 95% CI: -2.84, -1.15 g/week) (Fig. 2; Supplementary Table 5).

Among nulliparas, a similar pattern of association with an earlier starting gestational week was seen for HC growth pace (week 15 to 20), AC growth pace (week 18–38), HL growth pace (week 11–19, week 36–39), and FL growth pace (week 14–18, week 37–40). Among multiparas, only BPD growth pace was associated with mGRS at week 32–37, but the confidence intervals were wide (Supplementary Table 6). The results remained in sensitivity analyses with additional adjustment for maternal age and pre-pregnancy BMI (Supplementary Table 7). Moreover, exploratory analysis comparing nulliparas and primiparas (parity = 1) and primiparas and women with parity ≥ 2 showed that the association of mGRS with fetal growth measures weakens with increasing parity (Supplementary Table 8, Supplementary Fig. 1).

## Discussion

In a large ancestrally diverse pregnancy cohort with longitudinal ultrasound fetal measures, we found that increasing mGRS of lower birthweight was associated with decreases in fetal biometrics in nulliparous but not multiparous women. Our findings are strengthened by replication in another large independent cohort of nulliparas. The absence of association among multiparas despite a relatively larger sample size than nulliparas suggests that the maternal genetic influence on the intrauterine environment impacting fetal growth differs by parity. We also found that the gestational timing of association of mGRS with fetal growth in nulliparas differed by fetal biometry measure type. The earliest associations of mGRS were with fetal long skeletal bone (HL and FL) beginning at the first trimester, followed by association with HC at the second trimester, and the latest associations were with AC at the third trimester.

A key finding of our study was that mGRS was associated with fetal growth in nulliparous but not in multiparous women. This parity-related difference was consistent across fetal biometry measures. It has increasingly been recognized that nulliparous women face more constraints than multiparous women in meeting physiological adaptations of pregnancy, which are important to support fetal growth^50^. The most prominently known parity-related systemic differences are cardiovascular and hemodynamic changes associated with pregnancy. Nulliparous pregnant women have greater autonomic nervous system activity and less efficient balance of vascular tone, plasma volume, and cardiac output^51–54^. Corroborating this known phenomenon, the birthweight-lowering mGRS in our study included variants previously associated with hypertension and arterial pressure (e.g., *MTHFR* and *SH2B3*^55–60^). Therefore, it is possible that some of the maternal genetic variants linked to decreased fetal growth modulate cardiovascular processes during pregnancy, explaining the difference by parity.

Another vascular adaptation during early pregnancy that is known to differ between nulliparous and multiparous pregnancies is uteroplacental blood flow and invasiveness of placental trophoblast cells into the maternal uterine tissue^61^. Abnormal uterine artery flow and impaired trophoblast migration into the myometrium result in reduced placental surface, and decreased delivery of oxygen and nutrients, which contribute to decreased fetal growth^62,63^. Nulliparous women have lower uteroplacental blood flow rate^33,34^increased uterine vascular resistance^33^ and less extensive trophoblastic invasion of the decidual vessels^32,35^. Uteroplacental vascular resistance leads to hypoperfusion of the placenta and its downstream effects, including coagulation activation, endothelial cell dysfunction, placental thrombosis, and fibrin deposits, causing insufficient nutrients and oxygen supply to fetus, which is associated with lower fetal growth and development of growth restrictions^64–70^. In addition, multiparous women benefit from immunological memory of prior pregnancy experience, providing a protective tolerance that favors pregnancy outcomes^41^. Along these lines of evidence, our GRS included genes known to be associated with uterine artery notching and pre-eclampsia (e.g., *AGTR2* ^71^), immune traits (e.g., *ZBTB38* ^46,72^), metabolic, vascular, coagulation, and cardiac traits (Supplementary Table 9). Moreover, the genes annotating the SNPs in the GRS were significantly enriched for gene ontology biological process terms “immune response” and “hemostatic process” (Supplementary Table 10).

We found gestational timing differences in the association of maternal birthweight-lowering GRS with different fetal biometry measures in nulliparas. The gestational timings of association of GRS with fetal biometry largely coincided with previous reports on the timings of peak growth velocity of the fetal biometry measures^11^ and fetal body fat deposition (week 25 to term)^73–76^. To date, it remains unclear whether maternal GWAS loci associated with birthweight^4,7^ act on lean or fat mass, and at what gestational age. In our data, the *RREB1* variant showed a correlation with the growth pace of long skeletal bones before mid-gestation, and the *MTNR1B* variant is correlated with the pace of EFW after mid-gestation. These gestational periods are aligned with peak fetal growth velocity of the respective biometry measures^11^. *RREB1* is implicated in regulation of calcitonin, a component of skeletal bone which is elevated in blood of pregnant women^77^. *MTNR1B* is implicated in gestational diabetes, usually diagnosed in the third trimester of pregnancy^78^ and associated with birthweight and adiposity^79^. Metabolic and nutritional factors influence fetal fat mass primarily in later gestation, and calcium transport and hormones influence fat-free mass in earlier gestation ^80–82,83–85^. These studies and our findings suggest that the maternal genetic influence on birthweight is likely to result from a combination of its in-utero influence on long skeletal bone growth beginning in early gestation and on fat mass in later gestation.

We acknowledge the limitations of this study. Absence of fetal genotype data limited our ability to account for fetal genotypes in the analyses. To partly address this issue, the GRS excluded SNPs associated with birthweight through both maternal and fetal genomes. The distribution of parity as defined based on an obstetric criteria of reaching a specific gestational age is unclear in the birthweight GWAS studies. Genetic associations may not be generalizable across ancestral populations^86–88^. The extent to which the SNPs included in GRS, which were discovered in GWAS of birthweight in European ancestry populations, are generalizable to ancestrally diverse populations is unclear. Therefore, large GWAS studies of fetal biometry and neonatal measures involving ancestrally diverse populations are needed. Fetal growth trajectory in the replication cohort was modeled differently with two knots using ultrasound measurements at two study visits and birthweight to increase data points, which may show a slight shift from models using ultrasound measurements only.

In conclusion, we found that maternal birthweight-lowering GRS influences fetal growth among women giving birth to their first child but not in women giving birth to subsequent children. It is possible that the maternal genetic factors target major physiological changes at first pregnancy that get less profound with multi-parity. Consideration of parity as a biological variable may facilitate the precision of identifying sensitive intrauterine periods and interventions for pregnancy outcomes using genomics.

## Materials and methods

### Study populations and participants

The discovery analysis used data from the Eunice Kennedy Shriver National Institute of Child Health and Human Development (NICHD) Fetal Growth Studies-Singletons, which is a race/ethnic diverse cohort of pregnant women (*n* = 2802) enrolled between July 2009 and January 2013 at 12 participating clinical sites in the United States. Details on the inclusion and exclusion criteria, data collection and quality assurance, and ethical approval of the study have been reported before^47,89,90^. Briefly, the study recruited women aged 18 to 40 with a singleton pregnancy between 8 weeks + 0 days and 13 weeks + 6 days of gestation, had no major chronic medical conditions including diabetes, chronic hypertension or high blood pressure taking one or more medications for women with pre-pregnancy BMI < 30 kg/m^2^, and chronic hypertension or high blood pressure taking two or more medications for women with pre-pregnancy BMI ≥ 30 kg/m^2^. Women with pre-pregnancy BMI < 30 kg/m^2^ were also excluded if they conceived by ovulation stimulation drugs or assisted reproductive technology. After the first ultrasound to confirm gestational age, pregnant women were randomized to one of four groups for the purpose of scheduling visits to capture weekly fetal growth data. Women underwent up to five standardized ultrasounds with measurement of fetal biometry at a priori defined gestational ages. A total of 2802 women underwent ultrasound visits at 10–19 weeks, 1418 at 20–24 weeks, 1720 at 25–29 weeks, 2230 at 30–34 weeks, 2157 at 35–39 weeks, and 91 at 40 + weeks. Prior to obtaining measurements, all sonographers underwent training and credentialing and follow up assessments showed rigorous quality assurance^91,92^.

The replication analysis used data from the Nulliparous Pregnancy Outcome Study: Monitoring Mothers-to-be (nuMoM2b), which is a multi-ethnic cohort of nulliparas with singleton pregnancies (*n* = 10,038) recruited from hospitals affiliated with 8 clinical centers in the United States. Pregnant women were recruited if they had a viable singleton gestation, were between 6 weeks + 0 days and 13 weeks + 6 days’ gestation and had no previous pregnancy that lasted ≥ 20 weeks based on self-report and did not conceive using assisted reproduction with a donor oocyte. Study staff confirmed gestational age of any previous deliveries or pregnancy loss through a review of medical records, if available. Further details on the description of the nuMoM2b study, inclusion and exclusion criteria have been published previously^48,93^. Participants underwent three study visits during pregnancy (visit 1: 6 + 0 to 13 + 6 weeks, visit 2: 16 + 0 to 21 + 6 weeks, visit 3: 22 + 0 to 29 + 6 weeks) and a study visit at delivery. Ultrasound measurement of fetal biometry was performed at study visits 2 and 3. All sonographers for ultrasound studies were trained and certified.

The NICHD Fetal Growth Studies was approved by the Institutional Review Boards of NICHD and all participating clinical sites. The nuMoM2b study protocol was approved by each clinical site’s local governing Institutional Review Board. The study was conducted in accordance with the relevant guidelines and regulations. Written informed consent was obtained from all study participants in both cohorts.

### Fetal growth measures

In the NICHD Fetal Growth Studies-Singletons (discovery cohort), head circumference (HC), biparietal diameter (BPD), abdominal circumference (AC), humerus length (HL), and femur length (FL) were measured at each of the five ultrasound visits. Estimated fetal weight (EFW) was calculated from HC, AC, and FL using the Hadlock formula^94^. A linear mixed model, using a cubic spline mean structure and a cubic polynomial random effect structure with three knot points, was used to estimate the fetal growth trajectories per biometry across gestational age using log-transformed biometry measurement as the dependent variable^95^. Each estimated log-transformed biometry from the model was then exponentially transformed back to predict fetal size at weeks 10–40. Weekly fetal growth pace for each fetal biometry was calculated as the difference between the predicted fetal sizes of two consecutive weeks across 10–40 weeks of gestation.

In the nuMoM2b Study (replication cohort), ultrasound measurements of HC, BPD, AC, HL and FL were performed at visit 2 and 3^48,96^. EFW was calculated using the Hadlock formula. A linear mixed model, with a cubic spline mean structure with two knots and a cubic polynomial random effect structure, was used to create an EFW trajectory using EFW at the two visits and birthweight. The size and weekly pace of EFW were determined using the approach described above for the discovery cohort.

### Genotyping and quality control

For the discovery cohort, DNA was extracted from buffy coat of stored maternal blood specimens (*n* = 2215). Genome-wide single nucleotide polymorphisms (SNPs) were genotyped using the Infinium Multiethnic Global Bead Chip microarray (Illumina, California). Detailed quality control of genotypes and imputation procedures have been described previously^14^. A total of 2065 samples passed genotype quality control. After further filtration based on availability of data on two or more ultrasound measurements, fetal sex and parity, 1803 women (822 nulliparous, 981 multiparous) were included in the analyses. A total of 1803 women underwent ultrasound visits at 10–19 weeks, 968 at 20–24 weeks, 1175 at 25–29 weeks, 1553 at 30–34 weeks, 1524 at 35–39 weeks, and 71 at 40 + weeks.

In the replication cohort, DNA extracted from stored blood obtained from participating women (*n* = 9757) was genotyped using the Infinium Multi-Ethnic Global Array (Illumina, California). The quality control and imputation procedures have been detailed previously^97^. After exclusion of samples that failed genotype quality control, and with missing EFW, birthweight, gestational age at the two ultrasound visits or at birth, and fetal sex, 6042 nulliparous pregnant women were included in subsequent analyses.

### Selection of SNPs associated with birthweight via maternal-only effect

SNPs that are known to be genome-wide significantly associated with birthweight only through the maternal genome were selected from two published GWAS of birthweight in European ancestry populations^4,7^. A total of 37 SNPs with maternal-only effect on birthweight have been extracted from the two studies (i.e., 32 SNPs identified by Warrington et al. and 10 SNPs identified by Juliusdottir et al., of which five SNPs overlap in both studies). The reported effect coefficient (beta) and standard error values were extracted from each GWAS. For the five overlapping SNPs, the effect coefficient (beta) and standard error values were combined using inverse variance weighted meta-analysis implemented in METAL^98^. Out of the 37 SNPs, 32 SNPs available in our discovery cohort and 33 SNPs available in our replication cohort were included in further analyses (Supplementary Table 1).

### Calculation of genetic risk score

A genetic risk score (GRS) is an aggregated score of genetic variants associated with a phenotype^99^. For each woman, GRS of lower birthweight was calculated by multiplying the dosage (i.e., values range 0 to 2) of the birthweight decreasing allele of each candidate SNP by the corresponding effect coefficient (beta), followed by summing up the resulting values. GRS was then standardized by z-transformation across samples.

### Statistical analyses

Linear regression models adjusted for fetal sex and the first ten genetic principal components (PCs) were used to test the association of standardized GRS with each fetal biometry size (i.e., EFW, HC, BPD, AC, HL, and FL) at gestational weeks 10–40 and with each fetal biometry weekly pace (i.e., EFW, HC, BPD, HL, AC, and FL) at gestational weeks 11–40. Genotype-based PCs represent population stratification and were generated for each cohort using linkage disequilibrium (LD) pruned subset of SNPs (window size is 50, the number of SNPs to shift the window is 5, and R2 is 0.2) as implemented in PLINK v. 1.9 ^100^. All analyses were done separately for nulliparous and multiparous women in the discovery cohort. Similar analyses were performed for EFW in the replication cohort. Sensitivity analyses were performed for all models with additional adjustments for maternal pre-pregnancy body mass index (BMI) and age because of their correlation with parity. In exploratory analyses, we tested the association of GRS with each fetal biometry among primiparas (parity = 1; *n* = 642) and women with parity ≥ 2; *n* = 339), followed by a Welch’s t-test to compare the associations between nulliparas and primiparas, nulliparas and women with parity ≥ 2, and primiparas and women with parity ≥ 2. An association p-value < 0.05 was considered statistically significant.

## Electronic supplementary material

Below is the link to the electronic supplementary material.

Supplementary Material 1

Supplementary Material 2

Supplementary Material 3

Supplementary Material 4

Supplementary Material 5

Supplementary Material 6

Supplementary Material 7

Supplementary Material 8

Supplementary Material 9

Supplementary Material 10

Supplementary Material 11

## Data Availability

The genotype data underlying the findings reported in this study are available upon request through the NICHD Division of Intramural Population Health Research Biospecimen Repository Access and Data Sharing. nuMoM2b data can be accessed through dbGaP (accession phs002808.v1.p1).
