# Supplementary figures and images for: Maternal parity modifies the association of birthweight polygenic score with fetal growth
Source: Sci Rep. 2025 Jul 31;15:27915. doi: 10.1038/s41598-025-10415-1 (PMC12313924; doi:10.1038/s41598-025-10415-1)

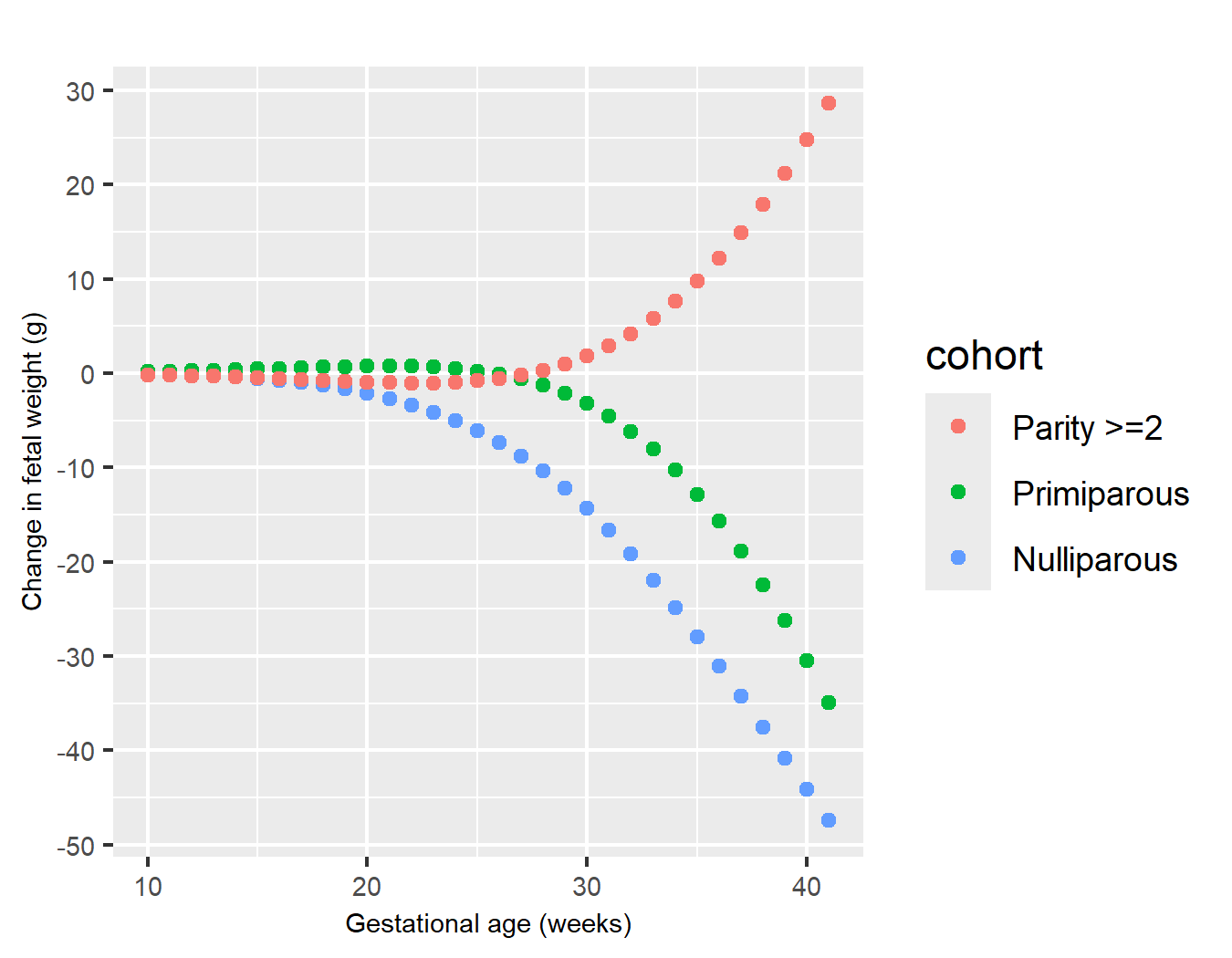

Supplement: Supplementary file 1 — Supplementary Material 1 [file 41598_2025_10415_MOESM1_ESM.tiff]
